# A Fluid Perspective of Relativistic Quantum Mechanics

**DOI:** 10.3390/e25111497

**Published:** 2023-10-30

**Authors:** Asher Yahalom

**Affiliations:** 1 Department of Electrical & Electronic Engineering, Faculty of Engineering, Ariel University, Ariel 40700, Israel; asya@ariel.ac.il; Tel.: +972-54-7740294; 2 Center for Astrophysics, Geophysics, and Space Sciences (AGASS), Ariel University, Ariel 40700, Israel

**Keywords:** spin, fluid dynamics, electromagnetic interaction

## Abstract

In previous papers, it has been shown how Schrödinger’s equation which includes an electromagnetic field interaction can be deduced from a fluid dynamical Lagrangian of a charged potential flow that interacts with an electromagnetic field. The quantum behaviour is derived from Fisher information terms added to the classical Lagrangian, showing that a quantum mechanical system is driven by information and not only electromagnetic fields. This program was applied to Pauli’s equations by removing the restriction of potential flow and using the Clebsch formalism. Although the analysis was quite successful, there were terms that did not admit interpretation, a number of which can be easily traced to the relativistic Dirac theory. Here, this analysis is repeated for a relativistic flow, pointing to a new approach for deriving relativistic quantum mechanics.

## 1. Introduction

A comprehensive introduction to the subject of the variational formalism of non-relativistic fluid dynamics and quantum mechanics and their deep interconnections is provided in [1,2], and we do not repeat it here.

The original work of Clebsch and all the following publications assume a non-relativistic fluid in which the velocity of the flow is much slower than the speed of light in a vacuum *c*. This is of course to be expected, as the work of Clebsch preceded Einstein’s work on special relativity by 48 years. There is a practical basis as well, as relativistic flows are hardly encountered on earth.

The standard approach to relativistic flows is based on the energy–momentum tensor [3,4,5]; however, this approach is not rigorous, as the definition of an energy–momentum tensor can only be carried out if a Lagrangian density is provided [6]. However, no Lagrangian density is known for relativistic flows. In this work, we intend to expand the work of Clebsch to relativistic flows and thereby amend this lacuna with a derived Lagrangian density for a relativistic flow, from which will be possible to obtain the rigorous energy–momentum tensor of high velocity flows.

We begin by introducing a variational principle for a relativistic charged classical particle with a vector potential interaction and a system of the same. This is followed by the Eckart [7] Lagrangian variational principles generalized for a relativistic charged fluid. We then introduce a Eulerian–Clebsch variational principle for a relativistic charged fluid. Finally, the concept of Fisher information allows us to suggest a new approach to relativistic quantum mechanics.

## 2. Variational Analysis of Relativistic Trajectories

We study a particle travelling in a constant metric spacetime. The action A for a relativistic particle is

(1) $$A=-mc\int d\tau -e\int A^{\alpha }dx_{\alpha }$$

where τ is the “length” (interval) along the trajectory

(2) $$d\tau^{2}=\left|\eta^{\alpha \beta }dx_{\alpha }dx_{\beta }\right|=\left|dx_{\alpha }dx^{\alpha }\right|$$

where $x_{\alpha }$ denotes the particle’s coordinates (the metric raises and lowers indices, as is customary), *m* denotes the particle’s mass, *e* denotes the particle’s charge, and $A^{\alpha }$ denotes the four-vector potential, which is a function of the particle coordinates. In addition, $A^{\alpha }$ transforms under Lorentz transformations as a four-dimensional vector. Variational analysis leads to the following equations:

(3) $$m\frac{du^{\alpha }}{d\tau }=-\frac{e}{c}u^{\beta }\left(\partial_{\beta }A^{\alpha }-\partial^{\alpha }A_{\beta }\right),\;u^{\alpha }\equiv \frac{dx^{\alpha }}{d\tau },\;\partial^{\alpha }\equiv \frac{\partial }{\partial x_{\alpha }},\;\partial_{\beta }\equiv \eta_{\beta \alpha }\partial^{\alpha }$$

in which the metric $\eta_{\alpha \beta }$ is the Lorentz–Minkowski metric:

(4) $$\eta_{\alpha \beta }=\;\mathrm{diag}\;\left(1,-1,-1,-1\right).$$

### 2.1. Space and Time

For a spacetime with a Lorentz–Minkowski metric, the partition into spatial and temporal coordinates is easy. The spatial coordinates are $\vec{x}=\left(x_{1},x_{2},x_{3}\right)$ (in which we introduce a three-dimensional vector notation) and the temporal coordinate is $x_{0}$. Time is measured in seconds and space in meters; thus, we need the conversion coefficient *c* to convert between the two system of units $x_{0}=ct$. We define both a three- and a four-dimensional velocity, as follows:

(5) $$\vec{v}\equiv \frac{d\vec{x}}{dt},\;v=\left|\vec{v}\right|,\;v_{\alpha }\equiv \frac{dx_{\alpha }}{dt}=\left(c,\vec{v}\right).$$

It customary to partition $A_{\alpha }$ into temporal and spatial pieces:

(6) $$A_{\alpha }=\left(A_{0},A_{1},A_{2},A_{3}\right)\equiv \left(A_{0},\vec{A}\right)\equiv \left(\frac{\phi }{c},\vec{A}\right)$$

where the factor $\frac{1}{c}$ preceding the scalar potential ϕ allows us to write the equations in MKS units, and as such is not needed for arbitrary types of units. We can now define a magnetic field

(7) $$\vec{B}=\vec{\nabla }\times \vec{A},$$

where $\vec{\nabla }$ is the nabla operator, and define the electric field as follows:

(8) $$\vec{E}=-\frac{\partial \vec{A}}{\partial t}-\vec{\nabla }\phi$$

If v<c, for the differential of the interval we obtain

(9) $$d\tau^{2}=c^{2}dt^{2}\left(1-\frac{v^{2}}{c^{2}}\right),\;d\tau =cdt\sqrt{1-\frac{v^{2}}{c^{2}}}=\frac{cdt}{\gamma },\;\gamma \equiv \frac{1}{\sqrt{1-\frac{v^{2}}{c^{2}}}}.$$

The non relativistic limit is v≪c, in which case:

(10) γ≃1.

From now on, we use the symbol ≃ for the low-velocity classical approximation (i.e., the non-relativistic limit). Thus, the spatial part of Equation (3) is obtained in the well-known form:

(11) $$\frac{d}{dt}\left(m\gamma \vec{v}\right)=\frac{d}{dt}\left(m\frac{\vec{v}}{\sqrt{1-\frac{v^{2}}{c^{2}}}}\right)=e\left(\vec{E}+\vec{v}\times \vec{B}\right),\;m\frac{d\vec{v}}{dt}≃e\left(\vec{E}+\vec{v}\times \vec{B}\right).$$

### 2.2. The Lagrangian

We can write the action (1) as a temporal integral, and thereby define the following Lagrangian:

(12) $$\begin{array}{ccc} A & = & \int_{t1}^{t2}Ldt,\;L=L_{0}+L_{i}\\ L_{0} & \equiv & -mc\frac{d\tau }{dt}=-\frac{mc^{2}}{\gamma }=-mc^{2}\sqrt{1-\frac{v^{2}}{c^{2}}}≃\frac{1}{2}mv^{2}-mc^{2},\\ L_{i} & \equiv & -eA^{\alpha }\frac{dx_{\alpha }}{dt}=e\left(\vec{A}\cdot \vec{v}-\phi \right). \end{array}$$

in the above, the ≃ symbol signifies a classical (low-speed) approximation. Note that the interaction part of the Lagrangian is the same for high and low speeds, while the kinetic part takes a different and simpler form for the low-speed cases.

### 2.3. The Action and Lagrangian for a System of Particles

Consider *N* particles indexed by n∈[1−N], with a corresponding mass $m_{n}$ and charge $e_{n}$. Each particle has a trajectory $x_{n}^{\alpha }\left(\tau_{n}\right)$, in which $\tau_{n}$ measures the interval already propagated along the trajectory; thus,

(13) $$u_{n}^{\alpha }\equiv \frac{dx_{n}^{\alpha }}{d\tau_{n}}.$$

**Figure 1:**
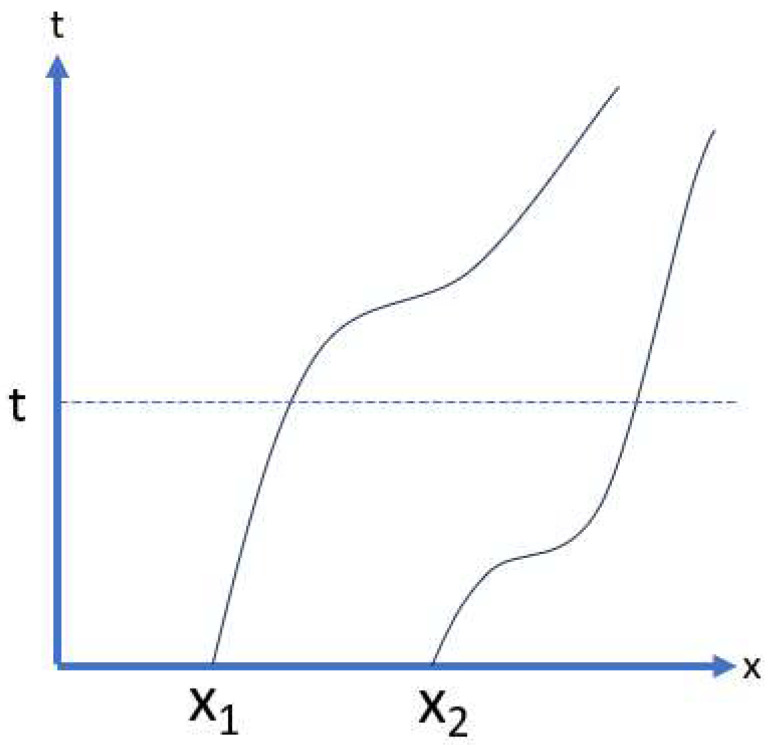
Schematic drawing of two trajectories piercing a time “plane”, which is illustrated as a straight line.

We assume as usual that the particle trajectories pierce through time “planes” and that the “plane” *t* is pierced at a position vector ${\vec{x}}_{n}\left(t\right)$; see Figure 1 (actually, each “plane” is three-dimensional).

Thus, we can define a velocity ${\vec{v}}_{n}\equiv \frac{d{\vec{x}}_{n}}{dt}$. The action and Lagrangian for every particle are as before:

(14) $$\begin{array}{ccc} A_{n} & = & -m_{n}c\int d\tau_{n}-e_{n}\int A^{\alpha }\left(x_{n}^{\nu }\right)dx_{\alpha n}=\int_{t1}^{t2}L_{n}dt,\;L_{n}\equiv L_{0n}+L_{in}\\ L_{0n} & \equiv & -\frac{m_{n}c^{2}}{\gamma_{n}}≃\frac{1}{2}m_{n}v_{n}^{2}-m_{n}c^{2},\;L_{in}\equiv e_{n}\left(\vec{A}\left({\vec{x}}_{n},t\right)\cdot {\vec{v}}_{n}-\phi \left({\vec{x}}_{n},t\right)\right). \end{array}$$

The action and Lagrangian for the system of particles are trivially deduced:

(15) $$A_{s}=\int_{t1}^{t2}L_{s}dt,\;L_{s}=\sum_{n=1}^{N}L_{n}.$$

The variational analysis follows the same route as for one particle, and the following set of equations are obtained:

(16) $$m_{n}\frac{du_{n}^{\alpha }}{d\tau_{n}}=-\frac{e_{n}}{c}u_{n}^{\beta }\left(\partial_{\beta }A_{n}^{\alpha }-\partial^{\alpha }A_{\beta n}\right),\;n\in \left[1-N\right].$$

For the three dimensional form,

(17) $$\frac{d}{dt}\left(\gamma_{n}{\vec{v}}_{n}\right)=\frac{e_{n}}{m_{n}}\left[{\vec{v}}_{n}\times \vec{B}\left({\vec{x}}_{n},t\right)+\vec{E}\left({\vec{x}}_{n},t\right)\right],\;n\in \left[1-N\right].$$

In the low velocity limit,

(18) $$\frac{d{\vec{v}}_{n}}{dt}≃\frac{e_{n}}{m_{n}}\left[{\vec{v}}_{n}\times \vec{B}\left({\vec{x}}_{n},t\right)+\vec{E}\left({\vec{x}}_{n},t\right)\right],\;n\in \left[1-N\right],$$

in which we do not sum over repeated Latin indices.

## 3. The Lagrangian Description of a Relativistic Charged Fluid

### 3.1. Action and Lagrangian

The dynamics of a flow are specified by its composition and the forces acting upon it. The fluid is composed of “fluid elements” [7,8]. A “fluid element” is a point particle with an infinitesimal mass $dM_{\vec{\alpha }}$, infinitesimal charge $dQ_{\vec{\alpha }}$, position four vector $x_{\vec{\alpha }\nu }\left(\tau_{\vec{\alpha }}\right)$, and $u_{\vec{\alpha }\nu }\left(\tau_{\vec{\alpha }}\right)\equiv \frac{dx_{\vec{\alpha }\nu }\left(\tau_{\vec{\alpha }}\right)}{d\tau_{\vec{\alpha }}}$. Here, the continuous vector label $\vec{\alpha }$ is present instead of the discrete index *n* used in the previous section. However, the “fluid element” is not a proper point particle, as it has an infinitesimal volume $dV_{\vec{\alpha }}$, infinitesimal entropy $dS_{\vec{\alpha }}$, and infinitesimal internal energy $dE_{in\;\vec{\alpha }}$. The action for each “fluid element” follows Equation (12) and has the form

(19) $$\begin{array}{ccc} & & dA_{\vec{\alpha }}=-dM_{\vec{\alpha }}c\int d\tau_{\vec{\alpha }}-dQ_{\vec{\alpha }}\int A^{\mu }\left(x_{\vec{\alpha }}^{\nu }\right)dx_{\mu \vec{\alpha }}+dA_{in\;\vec{\alpha }},\\ & & dA_{in\;\vec{\alpha }}\equiv -\int dE_{in\;\vec{\alpha }}dt. \end{array}$$

The Lagrangian for each “fluid element” can be derived from the above expression as follows:

(20) $$\begin{array}{ccc} dA_{\vec{\alpha }} & = & \int_{t1}^{t2}dL_{\vec{\alpha }}dt,\;dL_{\vec{\alpha }}\equiv dL_{k\vec{\alpha }}+dL_{i\vec{\alpha }}-dE_{in\;\vec{\alpha }}\\ dL_{k\vec{\alpha }} & \equiv & -\frac{dM_{\vec{\alpha }}c^{2}}{\gamma_{\vec{\alpha }}}≃\frac{1}{2}dM_{\vec{\alpha }}\;v_{\vec{\alpha }}{\left(t\right)}^{2}-dM_{\vec{\alpha }}c^{2}\\ dL_{i\vec{\alpha }} & \equiv & dQ_{\vec{\alpha }}\left(\vec{A}\left({\vec{x}}_{\vec{\alpha }}\left(t\right),t\right)\cdot {\vec{v}}_{\vec{\alpha }}\left(t\right)-\phi \left({\vec{x}}_{\vec{\alpha }}\left(t\right),t\right)\right). \end{array}$$

The action and Lagrangian of the entire fluid is integrated over all possible $\vec{\alpha }$’s:

(21) $$\begin{array}{ccc} L & = & \int_{\vec{\alpha }}dL_{\vec{\alpha }}\\ A & = & \int_{\vec{\alpha }}dA_{\vec{\alpha }}=\int_{\vec{\alpha }}\int_{t1}^{t2}dL_{\vec{\alpha }}dt=\int_{t1}^{t2}\int_{\vec{\alpha }}dL_{\vec{\alpha }}dt=\int_{t1}^{t2}Ldt. \end{array}$$

We define a density by dividing a fluid element quantity by its volume. This is done for the Lagrangian, mass, charge, and internal energy of every fluid element by introducing the following symbols:

(22) $$L_{\vec{\alpha }}\equiv \frac{dL_{\vec{\alpha }}}{dV_{\vec{\alpha }}},\;\rho_{\vec{\alpha }}\equiv \frac{dM_{\vec{\alpha }}}{dV_{\vec{\alpha }}},\;\rho_{c\vec{\alpha }}\equiv \frac{dQ_{\vec{\alpha }}}{dV_{\vec{\alpha }}},\;e_{in\;\vec{\alpha }}\equiv \frac{dE_{in\;\vec{\alpha }}}{dV_{\vec{\alpha }}}$$

Every quantity of the density type is a function of $\vec{x}$, in which the “fluid element” labelled $\vec{\alpha }$ is located in time *t*, for example,

(23) $$\rho \left(\vec{x},t\right)\equiv \rho \left({\vec{x}}_{\vec{\alpha }}\left(t\right),t\right)\equiv \rho_{\vec{\alpha }}\left(t\right).$$

In addition, we define “specific” quantities by dividing and the attribute of the “fluid element” by its mass, for example, a specific internal energy $\varepsilon_{\vec{\alpha }}$ is:

(24) $$\varepsilon_{\vec{\alpha }}\equiv \frac{dE_{in\;\vec{\alpha }}}{dM_{\vec{\alpha }}}\;\Rightarrow \;\rho_{\vec{\alpha }}\varepsilon_{\vec{\alpha }}=\frac{dM_{\vec{\alpha }}}{dV_{\vec{\alpha }}}\frac{dE_{in\;\vec{\alpha }}}{dM_{\vec{\alpha }}}=\frac{dE_{in\;\vec{\alpha }}}{dV_{\vec{\alpha }}}=e_{in\;\vec{\alpha }}.$$

Thus, we can partition the Lagrangian density as follows:

(25) $$\begin{array}{ccc} L_{\vec{\alpha }} & = & \frac{dL_{\vec{\alpha }}}{dV_{\vec{\alpha }}}=\frac{{dL}_{k\vec{\alpha }}}{dV_{\vec{\alpha }}}+\frac{{dL}_{i\vec{\alpha }}}{dV_{\vec{\alpha }}}-\frac{dE_{in\;\vec{\alpha }}}{dV_{\vec{\alpha }}}=L_{k\vec{\alpha }}+L_{i\vec{\alpha }}-e_{in\;\vec{\alpha }}\\ L_{k\vec{\alpha }} & \equiv & -\frac{\rho_{\vec{\alpha }}c^{2}}{\gamma_{\vec{\alpha }}}≃\frac{1}{2}\rho_{\vec{\alpha }}v_{\vec{\alpha }}{\left(t\right)}^{2}-\rho_{\vec{\alpha }}c^{2},\\ L_{i\vec{\alpha }} & \equiv & \rho_{c\vec{\alpha }}\left(\vec{A}\left({\vec{x}}_{\vec{\alpha }}\left(t\right),t\right)\cdot {\vec{v}}_{\vec{\alpha }}\left(t\right)-\varphi \left({\vec{x}}_{\vec{\alpha }}\left(t\right),t\right)\right). \end{array}$$

We can now write the fluid Lagrangian as a spatial integral:

(26) $$L=\int_{\vec{\alpha }}dL_{\vec{\alpha }}=\int_{\vec{\alpha }}L_{\vec{\alpha }}dV_{\vec{\alpha }}=\int L\left(\vec{x},t\right)d^{3}x$$

which we use in the later section concerned with the Eulerian representation of the fluid.

### 3.2. Variational Analysis

Here, we introduce the symbols $\Delta {\vec{x}}_{\vec{\alpha }}\equiv {\vec{\xi }}_{\vec{\alpha }}$ to denote a variation of the trajectory ${\vec{x}}_{\vec{\alpha }}\left(t\right)$, hence,

(27) $$\Delta {\vec{v}}_{\vec{\alpha }}\left(t\right)=\Delta \frac{d{\vec{x}}_{\vec{\alpha }}\left(t\right)}{dt}=\frac{d\Delta {\vec{x}}_{\vec{\alpha }}\left(t\right)}{dt}=\frac{d{\vec{\xi }}_{\vec{\alpha }}\left(t\right)}{dt},$$

and according to Equation (9),

(28) $$\Delta \left(\frac{1}{\gamma_{\vec{\alpha }}}\right)=-\frac{\gamma_{\vec{\alpha }}{\vec{v}}_{\vec{\alpha }}\left(t\right)}{c^{2}}\frac{d{\vec{\xi }}_{\vec{\alpha }}\left(t\right)}{dt},\;\Delta \gamma_{\vec{\alpha }}=\frac{\gamma_{\vec{\alpha }}^{3}{\vec{v}}_{\vec{\alpha }}\left(t\right)}{c^{2}}\frac{d{\vec{\xi }}_{\vec{\alpha }}\left(t\right)}{dt}.$$

An “ideal fluid” is defined by the fact that the “fluid element” does not exchange mass, electric charge, or heat with other elements; or in variational form,

(29) $$\Delta dM_{\vec{\alpha }}=\Delta dQ_{\vec{\alpha }}=\Delta dS_{\vec{\alpha }}=0.$$

According to thermodynamics, a change in the internal energy of a “fluid element” satisfies the below equation in the particle’s rest frame:

(30) $$\Delta dE_{in\;\vec{\alpha }0}=T_{\vec{\alpha }0}\Delta dS_{\vec{\alpha }0}-P_{\vec{\alpha }0}\Delta dV_{\vec{\alpha }0}.$$

In the above equation, the first term describes the heating of the “fluid element”, while the second term is a manifestation of the work performed by the “fluid element” on neighbouring elements; $T_{\vec{\alpha }0}$ denotes the temperature of the “fluid element” in the rest frame, and $P_{\vec{\alpha }0}$ is the pressure of the same. As the rest mass of the fluid element does not change and does not depend on any specific frame, we may divide the above expression by $dM_{\vec{\alpha }}$ to derive the variation of the specific energy:

(31) $$\begin{array}{ccc} \Delta \varepsilon_{\vec{\alpha }0} & = & \Delta \frac{dE_{in\;\vec{\alpha }0}}{dM_{\vec{\alpha }}}=T_{\vec{\alpha }0}\Delta \frac{dS_{\vec{\alpha }0}}{dM_{\vec{\alpha }}}-P_{\vec{\alpha }0}\Delta \frac{dV_{\vec{\alpha }0}}{dM_{\vec{\alpha }}}\\ & = & T_{\vec{\alpha }0}\Delta s_{\vec{\alpha }0}-P_{\vec{\alpha }0}\Delta \frac{1}{\rho_{\vec{\alpha }0}}=T_{\vec{\alpha }0}\Delta s_{\vec{\alpha }0}+\frac{P_{\vec{\alpha }0}}{\rho_{\vec{\alpha }0}^{2}}\Delta \rho_{\vec{\alpha }0}.\;s_{\vec{\alpha }0}\equiv \frac{dS_{\vec{\alpha }0}}{dM_{\vec{\alpha }}} \end{array}$$

where $s_{\vec{\alpha }0}$ is the specific entropy of the fluid element in its rest frame. It follows (suppressing the indices $\vec{\alpha }$ below) that

(32) $$\frac{\partial \varepsilon_{0}}{\partial s_{0}}=T_{0},\;\frac{\partial \varepsilon_{0}}{\partial \rho_{0}}=\frac{P_{0}}{\rho_{0}^{2}}.$$

Another useful thermodynamic quantity is the enthalpy, defined for a fluid element in its rest frame as follows:

(33) $$dW_{\vec{\alpha }0}=dE_{in\;\vec{\alpha }0}+P_{\vec{\alpha }0}dV_{\vec{\alpha }0}.$$

with the specific enthalpy then being

(34) $$w_{\vec{\alpha }0}=\frac{dW_{\vec{\alpha }0}}{dM_{\vec{\alpha }}}=\frac{dE_{in\;\vec{\alpha }0}}{dM_{\vec{\alpha }}}+P_{\vec{\alpha }0}\frac{dV_{\vec{\alpha }0}}{dM_{\vec{\alpha }}}=\varepsilon_{\vec{\alpha }0}+\frac{P_{\vec{\alpha }0}}{\rho_{\vec{\alpha }0}}.$$

Combining the above with Equation (32), we obtain the following useful property:

(35) $$w_{0}=\varepsilon_{0}+\frac{P_{0}}{\rho_{0}}=\varepsilon_{0}+\rho_{0}\frac{\partial \varepsilon_{0}}{\partial \rho_{0}}=\frac{\partial (\rho_{0}\varepsilon_{0})}{\partial \rho_{0}}.$$

Moreover,

(36) $$\frac{\partial w_{0}}{\partial \rho_{0}}=\frac{\partial (\varepsilon_{0}+\frac{P_{0}}{\rho_{0}})}{\partial \rho_{0}}=-\frac{P_{0}}{\rho_{0}^{2}}+\frac{1}{\rho_{0}}\frac{\partial P_{0}}{\partial \rho_{0}}+\frac{\partial \varepsilon_{0}}{\partial \rho_{0}}=-\frac{P_{0}}{\rho_{0}^{2}}+\frac{1}{\rho_{0}}\frac{\partial P_{0}}{\partial \rho_{0}}+\frac{P_{0}}{\rho_{0}^{2}}=\frac{1}{\rho_{0}}\frac{\partial P_{0}}{\partial \rho_{0}}.$$

For an ideal fluid, we neglect heat conduction and radiation, and consider only the convection. Thus, $\Delta dS_{\vec{\alpha }0}=0$, and it follows that:

(37) $$\Delta dE_{in\;\vec{\alpha }0}=-P_{0}\Delta dV_{\vec{\alpha }0}.$$

We now need to establish some relations between the rest frame and any other frame in which the fluid element is in motion; this frame is sometimes denoted the “laboratory” frame. First, note that in the rest frame there is (by definition) no velocity; hence, according to Equation (9), we have:

(38) $$d\tau =cdt_{0}=cdt\sqrt{1-\frac{v^{2}}{c^{2}}}=\frac{cdt}{\gamma }\;\Rightarrow \;dt_{0}=\frac{dt}{\gamma }.$$

It is well known that the four-volume is Lorentz invariant; hence,

(39) $$dV_{0}dt_{0}=dVdt=dVdt_{0}\gamma ,\;\Rightarrow dV_{0}=\gamma dV.$$

Thus,

(40) $$\rho_{0}=\frac{dM}{dV_{0}}=\frac{1}{\gamma }\frac{dM}{dV}=\frac{\rho }{\gamma },\;\Rightarrow \;\rho =\gamma \rho_{0}.$$

Moreover, the action provided in Equation (19) is Lorentz invariant; thus,

(41) $$dE_{in\;\vec{\alpha }0}dt_{0}=dE_{in\;\vec{\alpha }}dt=dE_{in\;\vec{\alpha }}dt_{0}\gamma \Rightarrow dE_{in\;\vec{\alpha }0}=\gamma dE_{in\;\vec{\alpha }},dE_{in\;\vec{\alpha }}=\frac{dE_{in\;\vec{\alpha }0}}{\gamma }.$$

We can now vary the internal energy of the fluid element:

(42) $$\Delta dE_{in\;\vec{\alpha }}=\Delta \left(\frac{1}{\gamma }\right)dE_{in\;\vec{\alpha }0}+\frac{1}{\gamma }\Delta dE_{in\;\vec{\alpha }0}.$$

Taking into account Equations (37) and (39), we obtain:

(43) $$\Delta dE_{in\;\vec{\alpha }}=\Delta \left(\frac{1}{\gamma }\right)dE_{in\;\vec{\alpha }0}-\frac{1}{\gamma }P_{0}\Delta dV_{\vec{\alpha }0}=\Delta \left(\frac{1}{\gamma }\right)dE_{in\;\vec{\alpha }0}-\frac{1}{\gamma }P_{0}\Delta \left(\gamma dV_{\vec{\alpha }}\right).$$

Thus, using the definition of enthalpy in Equation (33), we can write:

(44) $$\Delta dE_{in\;\vec{\alpha }}=\Delta \left(\frac{1}{\gamma }\right)\left(dE_{in\;\vec{\alpha }0}+P_{0}dV_{\vec{\alpha }0}\right)-\;P_{0}\Delta dV_{\vec{\alpha }}=\Delta \left(\frac{1}{\gamma }\right)dW_{\vec{\alpha }0}-\;P_{0}\Delta dV_{\vec{\alpha }}.$$

We now vary the volume element. At time *t*, the volume of the fluid element is:

(45) $$dV_{\vec{\alpha },t}=d^{3}x\left(\vec{\alpha },t\right).$$

The Jacobian relates this to the same element at t=0:

(46) $$d^{3}x\left(\vec{\alpha },t\right)=Jd^{3}x\left(\vec{\alpha },0\right),\;J\equiv {\vec{\nabla }}_{0}x_{1}\cdot \left({\vec{\nabla }}_{0}x_{2}\times {\vec{\nabla }}_{0}x_{3}\right)$$

where ${\vec{\nabla }}_{0}$ is calculated with respect to the t=0 coordinates of the fluid elements:

(47) $${\vec{\nabla }}_{0}\equiv \left(\frac{\partial }{\partial x{\left(\vec{\alpha },0\right)}_{1}},\frac{\partial }{\partial x{\left(\vec{\alpha },0\right)}_{2}},\frac{\partial }{\partial x{\left(\vec{\alpha },0\right)}_{3}}\right).$$

An interesting point is that the construction of the volume element of space from three vectors, for which we construct a Jacobian as in Equation (46), is very similar to the construction of a four-dimensional volume element from four scalars considering a Jacobian of the transformation of space time coordinates to scalar field space, as was done long ago in [9,10,11,12]. Thus,

(48) $$\begin{array}{ccc} \Delta dV_{\vec{\alpha },t} & = & \Delta d^{3}x\left(\vec{\alpha },t\right)=\Delta J\;d^{3}x\left(\vec{\alpha },0\right)=\frac{\Delta J}{J}d^{3}x\left(\vec{\alpha },t\right)=\frac{\Delta J}{J}dV_{\vec{\alpha },t},\\ (\Delta d^{3}x\left(\vec{\alpha },0\right) & = & 0). \end{array}$$

The variation of *J* can be derived as follows:

(49) $$\Delta J={\vec{\nabla }}_{0}\Delta x_{1}\cdot \left({\vec{\nabla }}_{0}x_{2}\times {\vec{\nabla }}_{0}x_{3}\right)+{\vec{\nabla }}_{0}x_{1}\cdot \left({\vec{\nabla }}_{0}\Delta x_{2}\times {\vec{\nabla }}_{0}x_{3}\right)+{\vec{\nabla }}_{0}x_{1}\cdot \left({\vec{\nabla }}_{0}x_{2}\times {\vec{\nabla }}_{0}\Delta x_{3}\right),$$

Now, we have:

(50) $$\begin{array}{ccc} & & {\vec{\nabla }}_{0}\Delta x_{1}\cdot \left({\vec{\nabla }}_{0}x_{2}\times {\vec{\nabla }}_{0}x_{3}\right)={\vec{\nabla }}_{0}\xi_{1}\cdot \left({\vec{\nabla }}_{0}x_{2}\times {\vec{\nabla }}_{0}x_{3}\right)\\ & = & \partial_{k}\xi_{1}{\vec{\nabla }}_{0}x_{k}\cdot \left({\vec{\nabla }}_{0}x_{2}\times {\vec{\nabla }}_{0}x_{3}\right)=\partial_{1}\xi_{1}{\vec{\nabla }}_{0}x_{1}\cdot \left({\vec{\nabla }}_{0}x_{2}\times {\vec{\nabla }}_{0}x_{3}\right)=\partial_{1}\xi_{1}J.\\ & & {\vec{\nabla }}_{0}x_{1}\cdot \left({\vec{\nabla }}_{0}\Delta x_{2}\times {\vec{\nabla }}_{0}x_{3}\right)={\vec{\nabla }}_{0}x_{1}\cdot \left({\vec{\nabla }}_{0}\xi_{2}\times {\vec{\nabla }}_{0}x_{3}\right)\\ & = & \partial_{k}\xi_{2}{\vec{\nabla }}_{0}x_{1}\cdot \left({\vec{\nabla }}_{0}x_{k}\times {\vec{\nabla }}_{0}x_{3}\right)=\partial_{2}\xi_{2}{\vec{\nabla }}_{0}x_{1}\cdot \left({\vec{\nabla }}_{0}x_{2}\times {\vec{\nabla }}_{0}x_{3}\right)=\partial_{2}\xi_{2}J.\\ & & {\vec{\nabla }}_{0}x_{1}\cdot \left({\vec{\nabla }}_{0}x_{2}\times {\vec{\nabla }}_{0}\Delta x_{3}\right)={\vec{\nabla }}_{0}x_{1}\cdot \left({\vec{\nabla }}_{0}x_{2}\times {\vec{\nabla }}_{0}\xi_{3}\right)\\ & = & \partial_{k}\xi_{3}{\vec{\nabla }}_{0}x_{1}\cdot \left({\vec{\nabla }}_{0}x_{2}\times {\vec{\nabla }}_{0}x_{k}\right)=\partial_{3}\xi_{3}{\vec{\nabla }}_{0}x_{1}\cdot \left({\vec{\nabla }}_{0}x_{2}\times {\vec{\nabla }}_{0}x_{3}\right)=\partial_{3}\xi_{3}J. \end{array}$$

Thus,

(51) $$\Delta J=\partial_{1}\xi_{1}J+\partial_{2}\xi_{2}J+\partial_{3}\xi_{3}J=\vec{\nabla }\cdot \vec{\xi }\;J,\;\Delta dV_{\vec{\alpha },t}=\vec{\nabla }\cdot \vec{\xi }\;dV_{\vec{\alpha },t}.$$

The variation of the internal energy of Equation (44) can be written as follows:

(52) $$\Delta dE_{in\;\vec{\alpha }}=\Delta \left(\frac{1}{\gamma }\right)dW_{\vec{\alpha }0}-P_{0}\vec{\nabla }\cdot \vec{\xi }\;dV_{\vec{\alpha },t}.$$

Taking into account Equation (28), this takes the following form:

(53) $$\Delta dE_{in\;\vec{\alpha }}=-P_{\vec{\alpha }0}\vec{\nabla }\cdot {\vec{\xi }}_{\vec{\alpha }}\;dV_{\vec{\alpha },t}-\frac{\gamma_{\vec{\alpha }}{\vec{v}}_{\vec{\alpha }}\left(t\right)}{c^{2}}dW_{\vec{\alpha }0}\cdot \frac{d{\vec{\xi }}_{\vec{\alpha }}\left(t\right)}{dt}.$$

The variation of internal energy is the only new calculation with respect to the calculation for a system of particles (as described previously) thus, the rest of the analysis is trivial. Varying Equation (19), we obtain

(54) $$\begin{array}{ccc} \Delta dA_{\vec{\alpha }} & = & \int_{t1}^{t2}\Delta dL_{\vec{\alpha }}dt,\;\Delta dL_{\vec{\alpha }}=\Delta dL_{k\vec{\alpha }}+\Delta dL_{i\vec{\alpha }}-\Delta dE_{in\;\vec{\alpha }}\\ \Delta dL_{k\vec{\alpha }} & = & -dM_{\vec{\alpha }}c^{2}\Delta \left(\frac{1}{\gamma_{\vec{\alpha }}}\right)=dM_{\vec{\alpha }}\gamma_{\vec{\alpha }}{\vec{v}}_{\vec{\alpha }}\left(t\right)\cdot \frac{d{\vec{\xi }}_{\vec{\alpha }}\left(t\right)}{dt},\\ \Delta dL_{i\vec{\alpha }} & = & dQ_{\vec{\alpha }}\left(\Delta \vec{A}\left({\vec{x}}_{\vec{\alpha }}\left(t\right),t\right)\cdot {\vec{v}}_{\vec{\alpha }}\left(t\right)+\vec{A}\left({\vec{x}}_{\vec{\alpha }}\left(t\right),t\right)\cdot \Delta {\vec{v}}_{\vec{\alpha }}\left(t\right)\right.\\ & - & \left.\Delta \phi ({\vec{x}}_{\vec{\alpha }}\left(t\right),t)\right). \end{array}$$

We can now combine the internal and kinetic parts of the varied Lagrangian, taking into account the specific enthalpy definition in Equation (34):

(55) $$\Delta dL_{k\vec{\alpha }}-\Delta dE_{in\;\vec{\alpha }}=dM_{\vec{\alpha }}\gamma_{\vec{\alpha }}\left(1+\frac{w_{0}}{c^{2}}\right){\vec{v}}_{\vec{\alpha }}\left(t\right)\cdot \frac{d{\vec{\xi }}_{\vec{\alpha }}\left(t\right)}{dt}+P_{\vec{\alpha }0}\vec{\nabla }\cdot {\vec{\xi }}_{\vec{\alpha }}\;dV_{\vec{\alpha },t}.$$

The electromagnetic interaction variation terms are not different than in the low-speed (non-relativistic) case (see for example Equations (A47) and (A48) of [1]), and their derivation is repeated here:

(56) $$d{\vec{F}}_{L\vec{\alpha }}\equiv dQ_{\vec{\alpha }}\left[{\vec{v}}_{\vec{\alpha }}\times \vec{B}\left({\vec{x}}_{\vec{\alpha }}\left(t\right),t\right)+\vec{E}\left({\vec{x}}_{\vec{\alpha }}\left(t\right),t\right)\right]$$

and

(57) $$\Delta dL_{i\vec{\alpha }}=\frac{d(dQ_{\vec{\alpha }}\vec{A}\left({\vec{x}}_{\vec{\alpha }}\left(t\right),t\right)\cdot {\vec{\xi }}_{\vec{\alpha }})}{dt}+d{\vec{F}}_{L\vec{\alpha }}\cdot {\vec{\xi }}_{\vec{\alpha }}.$$

We can introduce the shorthand notation

(58) $$\bar{\lambda }\equiv 1+\frac{w_{0}}{c^{2}},\;\lambda \equiv \gamma \bar{\lambda }=\gamma \left(1+\frac{w_{0}}{c^{2}}\right).$$

If the enthalpy of a fluid element in its rest frame is much smaller than its rest energy, we have:

(59) $$dW_{\vec{\alpha }0}\ll dM_{\vec{\alpha }}c^{2}\;\Rightarrow \;1\gg \frac{dW_{\vec{\alpha }0}}{dM_{\vec{\alpha }}c^{2}}=\frac{w_{\vec{\alpha }0}}{c^{2}}.$$

Thus, in the classical limit (which involves restrictions on the enthalpy of both the fluid element and its velocity), we have

(60) $$\bar{\lambda }≃1,\;\lambda ≃1.$$

The variation of the action of a relativistic fluid element is

(61) $$\begin{array}{ccc} \Delta dA_{\vec{\alpha }} & = & \int_{t1}^{t2}\Delta dL_{\vec{\alpha }}dt={\left.\left(dM_{\vec{\alpha }}\lambda_{\vec{\alpha }}{\vec{v}}_{\vec{\alpha }}\left(t\right)+dQ_{\vec{\alpha }}\vec{A}\left({\vec{x}}_{\vec{\alpha }}\left(t\right),t\right)\right)\cdot {\vec{\xi }}_{\vec{\alpha }}\right|}_{t1}^{t2}\\ & - & \int_{t1}^{t2}\left(dM_{\vec{\alpha }}\frac{d(\lambda_{\vec{\alpha }}{\vec{v}}_{\vec{\alpha }}\left(t\right))}{dt}\cdot {\vec{\xi }}_{\vec{\alpha }}-d{\vec{F}}_{L\vec{\alpha }}\cdot {\vec{\xi }}_{\vec{\alpha }}-P_{\vec{\alpha }0}\vec{\nabla }\cdot {\vec{\xi }}_{\vec{\alpha }}\;dV_{\vec{\alpha },t}\right)dt. \end{array}$$

Thus, the variation of the relativistic fluid action is

(62) $$\begin{array}{ccc} \Delta A & = & \int_{\vec{\alpha }}dA_{\vec{\alpha }}={\left.\int_{\vec{\alpha }}\left(dM_{\vec{\alpha }}\lambda_{\vec{\alpha }}{\vec{v}}_{\vec{\alpha }}\left(t\right)+dQ_{\vec{\alpha }}\vec{A}\left(\vec{x}\left(\vec{\alpha },t\right),t\right)\right)\cdot {\vec{\xi }}_{\vec{\alpha }}\right|}_{t1}^{t2}\\ & - & \int_{t1}^{t2}\int_{\vec{\alpha }}\left(dM_{\vec{\alpha }}\frac{d(\lambda_{\vec{\alpha }}{\vec{v}}_{\vec{\alpha }}\left(t\right))}{dt}\cdot {\vec{\xi }}_{\vec{\alpha }}-d{\vec{F}}_{L\vec{\alpha }}\cdot {\vec{\xi }}_{\vec{\alpha }}-P_{\vec{\alpha }0}\vec{\nabla }\cdot {\vec{\xi }}_{\vec{\alpha }}\;dV_{\vec{\alpha }}\right)dt. \end{array}$$

Now, according to Equation (22) we may write:

(63) $$dM_{\vec{\alpha }}=\rho_{\vec{\alpha }}\;dV_{\vec{\alpha }},\;dQ_{\vec{\alpha }}=\rho_{c\vec{\alpha }}\;dV_{\vec{\alpha }}$$

and using the above relations, we can turn the $\vec{\alpha }$ integral into a volume integral and write the variation of the fluid action, in which we suppress the $\vec{\alpha }$ labels:

(64) $$\Delta A={\left.\int \left(\rho \lambda \vec{v}+\rho_{c}\vec{A}\right)\cdot \vec{\xi }dV\right|}_{t1}^{t2}-\int_{t1}^{t2}\int \left(\rho \frac{d(\lambda \vec{v})}{dt}\cdot \vec{\xi }-{\vec{f}}_{L}\cdot \vec{\xi }-P_{0}\vec{\nabla }\cdot \vec{\xi }\right)dVdt.$$

In the above, we have introduced the Lorentz force density

(65) $${\vec{f}}_{L\vec{\alpha }}\equiv \frac{d{\vec{F}}_{L\vec{\alpha }}}{dV_{\vec{\alpha }}}=\rho_{c\vec{\alpha }}\left[{\vec{v}}_{\vec{\alpha }}\times \vec{B}\left({\vec{x}}_{\vec{\alpha }}\left(t\right),t\right)+\vec{E}\left({\vec{x}}_{\vec{\alpha }}\left(t\right),t\right)\right].$$

Now, because

(66) $$P_{0}\vec{\nabla }\cdot \vec{\xi }=\vec{\nabla }\cdot \left(P_{0}\vec{\xi }\right)-\vec{\xi }\cdot \vec{\nabla }P_{0},$$

and using the Gauss theorem, the variation of the action can be written as

(67) $$\begin{array}{ccc} \Delta A & = & {\left.\int \left(\rho \lambda \vec{v}+\rho_{c}\vec{A}\right)\cdot \vec{\xi }dV\right|}_{t1}^{t2}\\ & - & \int_{t1}^{t2}\left[\int \left(\rho \frac{d(\lambda \vec{v})}{dt}-{\vec{f}}_{L}+\vec{\nabla }P_{0}\right)\cdot \vec{\xi }dV-\oint P_{0}\vec{\xi }\cdot d\vec{\Sigma }\right]dt. \end{array}$$

It follows that the variation of the action vanishes for a $\vec{\xi }$ such that $\vec{\xi }\left(t1\right)=\vec{\xi }\left(t2\right)=0$ and is vanishing on a surface encapsulating the fluid, and otherwise is arbitrary only if the Euler equation for a relativistic charged fluid is satisfied, that is,

(68) $$\frac{d(\lambda \vec{v})}{dt}=-\frac{\vec{\nabla }P_{0}}{\rho }+\frac{{\vec{f}}_{L}}{\rho },\;\frac{d\vec{v}}{dt}≃-\frac{\vec{\nabla }P_{0}}{\rho }+\frac{{\vec{f}}_{L}}{\rho },$$

for the particular case where the fluid element is made up of identical microscopic particles each with a mass *m* and charge *e*. It follows that the mass and charge densities are proportional to the number density *n*:

(69) $$\rho =m\;n,\;\rho_{c}=e\;n\Rightarrow \frac{{\vec{f}}_{L}}{\rho }=k\left[\vec{v}\times \vec{B}+\vec{E}\right],k\equiv \frac{e}{m},$$

thus, except for the terms related to the internal energy, the equation is similar to that for a point particle. For a neutral fluid, we obtain the following form:

(70) $$\frac{d(\lambda \vec{v})}{dt}=-\frac{\vec{\nabla }P_{0}}{\rho },\;\frac{d\vec{v}}{dt}≃-\frac{\vec{\nabla }P_{0}}{\rho }.$$

Notably, some authors prefer to write the above equation in terms of the energy per element of the fluid per unit volume in the rest frame, which is the sum of the internal energy contribution and the rest mass contribution:

(71) $$e_{0}\equiv \rho_{0}c^{2}+\rho_{0}\varepsilon_{0}.$$

It is easy to show that:

(72) $$\bar{\lambda }=1+\frac{w_{0}}{c^{2}}=\frac{e_{0}+P_{0}}{\rho_{0}c^{2}}.$$

Using the above equality and a few manipulations, we may write Equation (70) in a form which is preferred by certain authors:

(73) $$\left(e_{0}+P_{0}\right)\frac{\gamma }{c^{2}}\frac{d(\gamma \vec{v})}{dt}=-\vec{\nabla }P_{0}-\frac{\gamma^{2}}{c^{2}}\frac{dP_{0}}{dt}\vec{v}.$$

In practical fluid dynamics, a fluid is described in terms of localized quantities instead of quantities related to unseen infinitesimal “fluid elements”. This is the Eulerian description of fluid dynamics, in which one uses flow fields rather than “fluid elements”, as is discussed further below.

## 4. The Clebsch Approach to a Relativistic Charged Eulerian Fluid

Here, we follow the derivation of [1,2,13], now taking into account the relativistic nature of the flow; this implies taking into account an action which is invariant under Lorentz transformations. Let us consider the following action:

(74) $$\begin{array}{ccc} A & \equiv & \int Ld^{3}xdt,\;L\equiv L_{0}+L_{2}+L_{i}\\ L_{0} & \equiv & -\rho \left(\frac{c^{2}}{\gamma }+\varepsilon \right)=-\rho_{0}\left(c^{2}+\varepsilon_{0}\right)=-e_{0},\;L_{2}\equiv \nu \partial^{\nu }\left(\rho_{0}u_{\nu }\right)-\rho_{0}\alpha u_{\nu }\partial^{\nu }\beta ,\\ L_{i} & \equiv & -\rho_{c}A^{\nu }v_{\nu },\;v_{\nu }\equiv \frac{dx_{\nu }}{dt}. \end{array}$$

In the non-relativistic limit, we can write

(75) $$L_{0}≃\rho \left(\frac{1}{2}v^{2}-\varepsilon -c^{2}\right),$$

taking into account that

(76) $$u_{\mu }=\gamma \left(c,\vec{v}\right)$$

and that $\rho =\gamma \rho_{0}$ according to Equation (40). Hence, it is easy to write the above Lagrangian densities in a space–time formalism:

(77) $$L_{2}=\nu \left[\frac{\partial \rho }{\partial t}+\vec{\nabla }\cdot \left(\rho \vec{v}\right)\right]-\rho \alpha \frac{d\beta }{dt}\;L_{i}=\rho_{c}\left(\vec{A}\cdot \vec{v}-\phi \right)$$

Here, we consider the variational variables to be functions of space and time (fields). These include a vector velocity field $\vec{v}\left(\vec{x},t\right)$ and density scalar field $\rho (\vec{x},t)$. The way to include conservation of quantities such as label, mass, charge and entropy in the above formalism, which is easy in the Lagrange approach, is to use Lagrange multipliers ν,α that enforce the following equations:

(78) $$\begin{array}{ccc} & & \frac{\partial \rho }{\partial t}+\vec{\nabla }\cdot \left(\rho \vec{v}\right)=0\\ & & \frac{d\beta }{dt}=0 \end{array}$$

Provided that ρ≠0, these are the continuity equation which ensures mass conservation and the condition that β be a label (co-moving function). Combining the variation with respect to β with the continuity Equation (78) leads to the following equation (similar to the derivation of Equations (67)–(69) of [2]):

(79) $$\frac{d\alpha }{dt}=0$$

Hence, for ρ≠0, both α and β are labels. The specific internal energy $\varepsilon_{0}$ defined in Equation (24) is dependent on the thermodynamic properties of the fluid. This is formulated through an equation of state as a function of the density and specific entropy. Here, we assume a barotropic fluid, that is, $\varepsilon_{0}\left(\rho_{0}\right)$ is a function of the density $\rho_{0}$ alone. The electromagnetic potentials $\vec{A},\phi$ are given functions of coordinates, and as such are not varied. Another assumption in our analysis is that each fluid element is composed of microscopic particles of mass *m* and charge *e*; thus, it follows from Equation (69) that

(80) $$\rho_{c}=k\rho .$$

We can now take the variational derivative with respect to the density ρ, obtaining:

(81) $$\begin{array}{ccc} \delta_{\rho }A & = & \int d^{3}xdt\delta \rho \left[-\frac{c^{2}}{\gamma }-w_{0}\frac{\delta \rho_{0}}{\delta \rho }-\frac{\partial \nu }{\partial t}-\vec{v}\cdot \vec{\nabla }\nu +k\left(\vec{A}\cdot \vec{v}-\phi \right)\right]\\ & + & \oint d\vec{S}\cdot \vec{v}\delta \rho \nu +\int d\vec{\Sigma }\cdot \vec{v}\delta \rho \left[\nu \right]+\int d^{3}{x\nu \delta \rho |}_{t_{0}}^{t_{1}} \end{array}$$

or:

(82) $$\begin{array}{ccc} \delta_{\rho }A & = & \int d^{3}xdt\delta \rho \left[-\frac{c^{2}+w_{0}}{\gamma }-\frac{\partial \nu }{\partial t}-\vec{v}\cdot \vec{\nabla }\nu +k\left(\vec{A}\cdot \vec{v}-\phi \right)\right]\\ & + & \oint d\vec{S}\cdot \vec{v}\delta \rho \nu +\int d\vec{\Sigma }\cdot \vec{v}\delta \rho \left[\nu \right]+\int d^{3}{x\nu \delta \rho |}_{t_{0}}^{t_{1}}. \end{array}$$

Hence, if δρ disappears on the boundary and cut, at the initial and final times we obtain:

(83) $$\frac{d\nu }{dt}=\frac{\partial \nu }{\partial t}+\vec{v}\cdot \vec{\nabla }\nu =-\frac{c^{2}+w_{0}}{\gamma }+k\left(\vec{A}\cdot \vec{v}-\phi \right)\Rightarrow \frac{d\nu }{dt}≃\frac{1}{2}v^{2}-w_{0}-c^{2}+k\left(\vec{A}\cdot \vec{v}-\phi \right).$$

In the above, we use the material derivative defined by the prevalent form:

(84) $$\frac{dg(\vec{\alpha },t)}{dt}=\frac{dg(\vec{x}\left(\vec{\alpha },t\right),t)}{dt}=\frac{\partial g}{\partial t}+\frac{d\vec{x}}{dt}\cdot \vec{\nabla }g=\frac{\partial g}{\partial t}+\vec{v}\cdot \vec{\nabla }g$$

when *g* is taken to be dependent on $\vec{x},t$. Equation (83) can be written in short form:

(85) $$\frac{d\nu }{dt}=-c^{2}\frac{\bar{\lambda }}{\gamma }+k\left(\vec{A}\cdot \vec{v}-\phi \right)=-c^{2}\frac{\lambda }{\gamma^{2}}+k\left(\vec{A}\cdot \vec{v}-\phi \right),$$

in which $\bar{\lambda }$ is defined in Equation (58). Finally, we vary the action with respect to $\vec{v}$, taking into account that

(86) $$\delta_{\vec{v}}\frac{1}{\gamma }=-\gamma \frac{\vec{v}\cdot \delta \vec{v}}{c^{2}}.$$

This results in

(87) $$\begin{array}{ccc} \delta_{\vec{v}}A & = & \int d^{3}xdt\rho \delta \vec{v}\cdot \left[\gamma \vec{v}-\frac{w_{0}}{\rho }\frac{\delta \rho_{0}}{\delta \vec{v}}-\vec{\nabla }\nu -\alpha \vec{\nabla }\beta +k\vec{A}\right]\\ & + & \oint d\vec{S}\cdot \delta \vec{v}\rho \nu +\int d\vec{\Sigma }\cdot \delta \vec{v}\rho \left[\nu \right], \end{array}$$

however,

(88) $$\frac{\delta \rho_{0}}{\delta \vec{v}}=\rho \frac{\delta \frac{1}{\gamma }}{\delta \vec{v}}=-\rho \gamma \frac{\vec{v}}{c^{2}}.$$

Taking in account the definition of λ (see Equation (58)), we have

(89) $$\begin{array}{ccc} \delta_{\vec{v}}A & = & \int d^{3}xdt\rho \delta \vec{v}\cdot \left[\lambda \vec{v}-\vec{\nabla }\nu -\alpha \vec{\nabla }\beta +k\vec{A}\right]\\ & + & \oint d\vec{S}\cdot \delta \vec{v}\rho \nu +\int d\vec{\Sigma }\cdot \delta \vec{v}\rho \left[\nu \right]. \end{array}$$

The above boundary terms contain an integration over the external boundary $\oint d\vec{S}$ and an integral over the cut $\int d\vec{\Sigma }$ that must be introduced in the case that ν is not single-valued; more details on this case are provided in the later sections of this paper. The external boundary term vanishes in the case of astrophysical flows for which ρ=0 on the free flow boundary or the case in which the fluid is contained in a vessel which induces a no-flux boundary condition $\delta \vec{v}\cdot \hat{n}=0$ (where $\hat{n}$ is a unit vector normal to the boundary). The cut “boundary” term vanishes when the velocity field varies only parallel to the cut, that is, it satisfies a Kutta-type condition. If the boundary terms vanish, then $\vec{v}$ must have the following form:

(90) $$\lambda \vec{v}=\alpha \vec{\nabla }\beta +\vec{\nabla }\nu -k\vec{A},\Rightarrow \vec{v}≃\alpha \vec{\nabla }\beta +\vec{\nabla }\nu -k\vec{A},$$

which is a generalization of the Clebsch representation of the flow field (see for example [7] and [14], p. 248) for a relativistic charged flow. Note that the ν function contributes to the velocity field through its gradient; this means that we can add any function of time to ν without altering the physical field. Redefining

(91) $$\bar{\nu }\equiv \nu +c^{2}t,$$

we obtain the more standard classical form of Equations (83) and (90):

(92) $$\frac{d\bar{\nu }}{dt}≃\frac{1}{2}v^{2}-w_{0}+k\left(\vec{A}\cdot \vec{v}-\phi \right),\;\vec{v}≃\alpha \vec{\nabla }\beta +\vec{\nabla }\bar{\nu }-k\vec{A}.$$

### 4.1. Euler’s Equations

We now show that a velocity field provided by Equation (90) such that the functions α,β,ν satisfy the corresponding Equations (78), (79) and (83) must satisfy Euler’s equations. Let us first calculate the material derivative of $\lambda \vec{v}$:

(93) $$\frac{d(\lambda \vec{v})}{dt}=\frac{d\vec{\nabla }\nu }{dt}+\frac{d\alpha }{dt}\vec{\nabla }\beta +\alpha \frac{d\vec{\nabla }\beta }{dt}-k\frac{d\vec{A}}{dt}.$$

It can be easily shown that:

(94) $$\begin{array}{ccc} \frac{d\vec{\nabla }\nu }{dt} & = & \vec{\nabla }\frac{d\nu }{dt}-\vec{\nabla }v_{n}\frac{\partial \nu }{\partial x_{n}}=\vec{\nabla }\left(-\frac{c^{2}+w_{0}}{\gamma }+k\vec{A}\cdot \vec{v}-k\phi \right)-\vec{\nabla }v_{n}\frac{\partial \nu }{\partial x_{n}}\\ \frac{d\vec{\nabla }\beta }{dt} & = & \vec{\nabla }\frac{d\beta }{dt}-\vec{\nabla }v_{n}\frac{\partial \beta }{\partial x_{n}}=-\vec{\nabla }v_{n}\frac{\partial \beta }{\partial x_{n}} \end{array}$$

in which $x_{n}$ is a Cartesian coordinate and a summation convention is assumed. Inserting the result from Equation (94) into Equation (93) yields:

(95) $$\begin{array}{ccc} & & \frac{d(\lambda \vec{v})}{dt}=-\vec{\nabla }v_{n}\left(\frac{\partial \nu }{\partial x_{n}}+\alpha \frac{\partial \beta }{\partial x_{n}}\right)+\vec{\nabla }\left(-\frac{c^{2}+w_{0}}{\gamma }+k\vec{A}\cdot \vec{v}-k\phi \right)-k\frac{d\vec{A}}{dt}\\ & = & -\vec{\nabla }v_{n}\left(\lambda v_{n}+kA_{n}\right)+\vec{\nabla }\left(-\frac{c^{2}+w_{0}}{\gamma }+k\vec{A}\cdot \vec{v}-k\phi \right)-k\partial_{t}\vec{A}-k\left(\vec{v}\cdot \vec{\nabla }\right)\vec{A}\\ & = & -\frac{1}{\gamma }\vec{\nabla }w_{0}+k\vec{E}+k\left(v_{n}\vec{\nabla }A_{n}-v_{n}\partial_{n}\vec{A}\right), \end{array}$$

where the electric field is defined in Equation (8). Furthermore, according to Equation (7),

(96) $${\left(v_{n}\vec{\nabla }A_{n}-v_{n}\partial_{n}\vec{A}\right)}_{l}=v_{n}\left(\partial_{l}A_{n}-\partial_{n}A_{l}\right)=\epsilon_{lnj}v_{n}B_{j}={\left(\vec{v}\times \vec{B}\right)}_{l},$$

hence, we obtain the Euler equation of a charged relativistic fluid in the following form:

(97) $$\frac{d(\lambda \vec{v})}{dt}=-\frac{1}{\gamma }\vec{\nabla }w_{0}+k\left[\vec{v}\times \vec{B}+\vec{E}\right]=-\frac{1}{\rho }\vec{\nabla }P_{0}+k\left[\vec{v}\times \vec{B}+\vec{E}\right],$$

which is due to (see Equation (36)):

(98) $$\vec{\nabla }w_{0}=\frac{\partial w_{0}}{\partial \rho_{0}}\vec{\nabla }\rho_{0}=\frac{1}{\rho_{0}}\frac{\partial P_{0}}{\partial \rho_{0}}\vec{\nabla }\rho_{0}=\frac{1}{\rho_{0}}\vec{\nabla }P_{0}.$$

Equation (97) is identical to Equation (68); thus, it is proven that the Euler equations can be derived from the action (77), meaning that all the equations of relativistic charged fluid dynamics can be derived from the action in (77) using arbitrary and unrestricted variations.

### 4.2. Simplified Action

It may be claimed the previous approach introduces unnecessary complications to the theory of relativistic fluid dynamics by adding three additional scalar fields α,β,ν to the physical set $\vec{v},\rho$. We show that this is just a superficial impression, and that Equation (74) in the pedagogical form can be further simplified. It is easy to show that, defining a four-dimensional Clebsch four vector:

(99) $$v_{C}^{\mu }\equiv \alpha \partial^{\mu }\beta +\partial^{\mu }\nu =\left(\frac{1}{c}\left(\alpha \partial_{t}\beta +\partial_{t}\nu \right),\alpha \vec{\nabla }\beta +\vec{\nabla }\nu \right)=\left(\frac{1}{c}\left(\alpha \partial_{t}\beta +\partial_{t}\nu \right),{\vec{v}}_{C}\right)$$

and a four dimensional electromagnetic Clebsch four vector:

(100) $$v_{E}^{\mu }\equiv v_{C}^{\mu }+kA^{\mu }=\left(v_{E}^{0},{\vec{v}}_{E}\right)=\left(\frac{1}{c}\left(\alpha \partial_{t}\beta +\partial_{t}\nu +k\phi \right),{\vec{v}}_{C}-k\vec{A}\right),$$

it follows from Equations (78), (85) and (90) that:

(101) $$v_{\mu }=-\frac{v_{E\mu }}{\lambda }\Rightarrow \;\vec{v}=\frac{{\vec{v}}_{E}}{\lambda },\;\lambda =-\frac{1}{c^{2}}\left(\alpha \partial_{t}\beta +\partial_{t}\nu +k\phi \right).$$

The classical limit of the above equations is

(102) $$\vec{v}≃{\vec{v}}_{E},\;\lambda ≃1.$$

Eliminating the Lagrangian density $\vec{v}$ appearing in Equation (77), the equation can be written (up to surface terms) in the following compact form:

(103) $$\hat{L}\left[\rho_{0},\alpha ,\beta ,\nu \right]=\rho_{0}\left[c\sqrt{v_{E\mu }v_{E}^{\mu }}-\varepsilon_{0}-c^{2}\right]$$

This Lagrangian density yields the four Equations in (78), (79), and (83); after these equations are solved, we can substitute the scalar fields α,β,ν into Equation (90) to obtain $\vec{v}$. Hence, the general charged relativistic barotropic fluid dynamics problem is modified such that instead of solving the Euler and continuity equations we need to solve an alternative equivalent set, which can be derived from the Lagrangian density $\hat{L}$. The classical limit of the Equation (103) can be calculated as follows. First, note that

(104) $$\sqrt{v_{E\mu }v_{E}^{\mu }}=\left|v_{E0}\right|\sqrt{1-\frac{{\vec{v}}_{E}^{2}}{v_{E0}^{2}}}=\left|v_{E0}\right|\sqrt{1-\frac{{\vec{v}}^{2}}{c^{2}}}=\frac{|v_{E0}|}{\gamma },$$

in which we have used Equation (101). According to Equation (101), $v_{E0}<0$, as λ>0; thus,

(105) $$|v_{E0}|=-v_{E0}=-\frac{1}{c}\left(\alpha \partial_{t}\beta +\partial_{t}\nu +k\phi \right).$$

Combining Equation (104) and Equation (105), it follows that

(106) $$c\sqrt{v_{E\mu }v_{E}^{\mu }}=-\frac{1}{\gamma }\left(\alpha \partial_{t}\beta +\partial_{t}\nu +k\phi \right),$$

which can be written in term of $\bar{\nu }$, defined in Equation (91) as follows:

(107) $$c\sqrt{v_{E\mu }v_{E}^{\mu }}=-\frac{1}{\gamma }\left(\alpha \partial_{t}\beta +\partial_{t}\bar{\nu }+k\phi -c^{2}\right).$$

We can now write Equation (103) in the following form:

(108) $$\begin{array}{ccc} \hat{L} & = & \rho_{0}\left[-\frac{1}{\gamma }\left(\alpha \partial_{t}\beta +\partial_{t}\bar{\nu }+k\phi -c^{2}\right)-\varepsilon_{0}-c^{2}\right]\\ & = & -\frac{\rho_{0}}{\gamma }\left[\alpha \partial_{t}\beta +\partial_{t}\bar{\nu }+k\phi +\gamma \varepsilon_{0}+\left(\gamma -1\right)c^{2}\right]. \end{array}$$

The classical limit $\frac{v}{c}\to 0,\gamma \to 1$ is straightforward, except for the last term, which should be handled with care:

(109) $$\left(\gamma -1\right)c^{2}=\left(\frac{\gamma -1}{{\left(\frac{v}{c}\right)}^{2}}\right)v^{2}≃\frac{1}{2}v^{2}≃\frac{1}{2}v_{E}^{2}.$$

Taking into account Equation (109), it follows that

(110) $$\hat{L}≃-\rho_{0}\left[\frac{\partial \bar{\nu }}{\partial t}+\alpha \frac{\partial \beta }{\partial t}+\varepsilon \left(\rho_{0}\right)+k\phi +\frac{1}{2}{\left(\vec{\nabla }\bar{\nu }+\alpha \vec{\nabla }\beta -k\vec{A}\right)}^{2}\right].$$

However, from Equation (41) it follows that

(111) $$\varepsilon_{0}=\frac{dE_{in\;\vec{\alpha }0}}{dM_{\vec{\alpha }}}=\gamma \frac{dE_{in\;\vec{\alpha }}}{dM_{\vec{\alpha }}}=\gamma \varepsilon .$$

Thus, in the classical limit there is no difference between ρ and $\rho_{0}$ on the one hand and $\varepsilon_{0}$ and ε on the other, as the difference is of order ${\left(\frac{v}{c}\right)}^{2}$. Therefore, we may write

(112) $$\hat{L}≃-\rho \left[\frac{\partial \bar{\nu }}{\partial t}+\alpha \frac{\partial \beta }{\partial t}+\varepsilon \left(\rho \right)+k\phi +\frac{1}{2}{\left(\vec{\nabla }\bar{\nu }+\alpha \vec{\nabla }\beta -k\vec{A}\right)}^{2}\right],$$

which is exactly the same classical Lagrangian density provided in Equation (20) of [1] (with $\bar{\nu }$ replacing ν and ϕ replacing φ).

## 5. Conclusions

While the analogies between spin fluid dynamics and classical Clebsch fluid dynamics are quite convincing [1,2], there are terms in spin fluid dynamics that lack a classical interpretation. Thus, it has been suggested that these terms originate from a relativistic Clebsch theory, which was the main motivation for the current paper. Indeed, following in the footsteps of previous papers [15,16], we may replace the internal energy in Equation (103) with a Lorentz-invariant Fisher information term to obtain a new Lagrangian density of the relativistic quantum mechanics of a particle with spin:

(113) $$L\left[\rho_{0},\alpha ,\beta ,\nu \right]=\rho_{0}\left[c\sqrt{v_{E\mu }v_{E}^{\mu }}-c^{2}\right]+\frac{\hbar^{2}}{2m}\partial^{\mu }a_{0}\partial_{\mu }a_{0},\;a_{0}\equiv \sqrt{\frac{\rho_{0}}{m}}$$

where *m* is the particle’s mass and *ℏ* is Planck’s constant divided by 2π.

A side benefit of the above work is the ability to canonically derive the momentum energy tensor of a relativistic fluid.

As the current paper is of limited scope, we have not been able to compare the above Lagrangian with its low-speed limit and derive the relevant quantum equation; hopefully, this will be done in a subsequent more expanded paper. However, it is easy to see even here that Schrödinger’s theory is the limit of the theory described by the Lagrangian density of Equation (113). We write the last term of this equation explicitly as

(114) $$\frac{\hbar^{2}}{2m}\partial^{\mu }a_{0}\partial_{\mu }a_{0}=\frac{\hbar^{2}}{2m}\left(\frac{1}{c^{2}}{\left(\partial_{t}a_{0}\right)}^{2}-{\left(\vec{\nabla }a_{0}\right)}^{2}\right).$$

If $\frac{1}{c}\left|\partial_{t}a_{0}\right|\ll \left|\vec{\nabla }a_{0}\right|$, which is a classical limit, as it requires that the amplitude typical gradient length over the typical amplitude changing time to be much smaller than *c*, we may write

(115) $$\frac{\hbar^{2}}{2m}\partial^{\mu }a_{0}\partial_{\mu }a_{0}≃-\frac{\hbar^{2}}{2m}{\left(\vec{\nabla }a_{0}\right)}^{2}.$$

The first terms of Equation (113) can be classically approximated through Equation (112), in which we assume no internal energy ε=0 and no vorticity α=β=0; thus, we obtain

(116) $$L≃-\rho \left[\frac{\partial \bar{\nu }}{\partial t}+k\phi +\frac{1}{2}{\left(\vec{\nabla }\bar{\nu }-k\vec{A}\right)}^{2}\right]-\frac{\hbar^{2}}{2m}{\left(\vec{\nabla }a_{0}\right)}^{2},$$

which is identical to the Schrödinger’s Lagrangian density (45) in [1] with a minor change of notation: $\rho \to \hat{\rho },\bar{\nu }\to \hat{\nu },\phi \to \varphi ,a_{0}\to a$. Thus, Schrödinger’s quantum theory is a classical limit of the relativistic quantum theory represented by Equation (113).

We conclude by acknowledging the importance of the comparison between the fluid route to relativistic quantum mechanics and the more established route laid out by Dirac; this certainly deserves an additional paper, which I hope to compose in the near future. 

## Data Availability

Data sharing not applicable.
